# Dietary transition proxies and ischemic heart disease burden: a country-year panel analysis with a systematic robustness assessment

**DOI:** 10.3389/fnut.2026.1883097

**Published:** 2026-08-18

**Authors:** Moon-Yeon Oh

**Affiliations:** Seoul St. Mary’s Hospital, College of Medicine, The Catholic University of Korea, Seoul, Republic of Korea

**Keywords:** country-year panel, dietary transition, ecological study, Global Burden of Disease, ischemic heart disease, sensitivity analysis, trans-fatty acids

## Abstract

Dietary trans fatty acids (TFA) are established risk factors for ischemic heart disease (IHD) at the individual level, but the robustness of country-level ecological associations across dietary transition proxies has rarely been examined systematically. We constructed a country-year ecological panel (8,536 country-years; 194 countries; 1980–2023) linking GBD 2021 total trans-fat exposure, GBD 2023 IHD/stroke outcomes, FAOSTAT dietary supply proxies, WHO policy indicators, and World Bank covariates. Two-way fixed-effects regressions with country-clustered standard errors were estimated at 0-, 3-, 5-, and 10-year lags and subjected to a systematic robustness program: alternative exposure constructions, population weighting, leave-one-country-out refitting, sample-composition exclusions, formal tests of income- and country-size interaction, and multiple-comparison adjustment. At the 5-year lag, higher estimated total trans-fat exposure was associated with higher IHD incidence (*β* = 5.77 per 0.1% point energy/day; 95% CI: 1.29–10.26; *p* = 0.012). This association was the most robust examined: it held in all 186 leave-one-country-out refits and across simple, population-weighted, and age-standardized exposure constructions (r ≥ 0.9997) and across OLS and Gamma GLM. However, it could not be separated from the development transition. The unadjusted two-way fixed-effects association remained significant after excluding island-states, whereas the fully covariate-adjusted estimate in the island-excluded sample was attenuated to non-significance (*p* = 0.111, *β* = 3.51). Sequential adjustment localized this attenuation to entanglement with urbanization and adiposity rather than to island states. Weighting each observation by population produced null but underpowered estimates (about 39% power to detect the unweighted effect; effective sample 12.9 of 186 countries), with confidence intervals containing the unweighted estimate. Formal interaction tests showed no significant modification by income group or, for IHD incidence, by country size. Sugar/sweeteners supply was positively associated with IHD incidence (*β* = 3.27; 95% CI: 0.25–6.29; *p* = 0.034) but did not survive multiple-comparison adjustment. This panel documents a positive trans-fat/IHD association that is consistent across estimators and exposure constructions and at lag windows up to 5 years. However, it is entangled with the development transition, cannot be isolated from it, and could be explained by modest unmeasured confounding (E-values 1.15–1.20); the association is therefore hypothesis-generating and does not, on its own, support causal or policy conclusions.

## 1 Introduction

Ischemic heart disease (IHD) is the leading single cause of death and disability-adjusted life years (DALYs) worldwide, accounting for more than 9 million deaths annually (1, 2). The country-level IHD burden varies by more than tenfold across regions, reflecting differences in diet, lifestyle, healthcare access, and socioeconomic development (1). Rapid dietary transition, marked by rising consumption of ultra-processed foods, refined carbohydrates, added sugars, and industrially modified fats, has coincided with increasing IHD burden in low- and middle-income countries (LMICs) (3, 4). Identifying which components of this dietary transition drive the population-level IHD burden is central to cardiovascular prevention policy (5).

Dietary guidance for IHD prevention has evolved considerably. Trans-fatty acids (TFA) emerged as a particularly harmful component: TFA increases LDL cholesterol, decreases HDL cholesterol, promotes vascular inflammation, and has consistently been associated with coronary heart disease risk at the individual level (6–8). Industrially produced trans fatty acids (TFA), formed by partial hydrogenation of vegetable oils, are the dominant source in most food supplies (9), and the World Health Organization (WHO) has called for their global elimination through the REPLACE initiative (10, 11). Ruminant TFA, present in dairy and meat, has not shown comparable harmful associations (6). Importantly for the present study, the GBD dietary risk exposure used here quantifies total trans-fat from all sources rather than the industrially produced fraction alone (Section 2.2). Simultaneously, refined carbohydrates, added sugars, and ultra-processed foods have become increasingly prominent worldwide, including in rapidly transitioning LMICs (3, 4, 12). Added sugar intake has been associated with CVD mortality (13–15), and carbohydrate quality has been identified as an important dimension of IHD risk (16). Country-level supply of seed oils and processed-food ingredients should be interpreted in this food system context, as indicators of dietary transition and industrial food penetration, rather than as direct evidence of specific fatty acid effects (17, 18).

Previous country-level analyses have examined dietary fat supply or trans-fat exposure in relation to cardiovascular mortality; however, many relied on total CVD outcomes, cross-sectional designs, or limited dietary comparators (19). A particularly relevant methodological precedent is a multi-country fixed-effect panel analysis of palm oil consumption and IHD/stroke mortality from 1980 to 1997, which linked national palm oil consumption to WHO mortality outcomes and reported stronger positive associations with IHD mortality in developing countries (20). Our study extends this country-level panel framework by using contemporary GBD IHD incidence, mortality, and DALY outcomes, evaluating multiple lag windows, and comparing trans-fat exposure against multiple dietary transition proxies, including seed oils, animal fats, wheat, and sugar/sweeteners, rather than focusing on a single dietary component. Total CVD combines heterogeneous outcomes with differing dietary aetiologies; focusing on the IHD endpoint targets a more aetiologically homogeneous outcome.

Ecological country-level analyses of this kind are, however, vulnerable to sample composition, exposure-construction choices, analytical weighting, and multiplicity, and these vulnerabilities are rarely examined systematically. We therefore treat the robustness assessment as a co-equal aim rather than an appendix. This study had three objectives: (1) to estimate country-level associations between trans-fat exposure and IHD incidence, deaths, and DALYs at multiple lag periods, and to compare them with ischemic stroke and total CVD as comparator endpoints; (2) to compare IHD associations across trans-fat, seed-oil, animal-fat, butter/ghee, wheat, and sugar/sweetener proxies as indicators of dietary transition; and (3) to establish, through a systematic robustness program—alternative exposure constructions, population weighting, leave-one-country-out refitting, sample-composition exclusions, formal interaction testing, and multiple-comparison adjustment—which of these associations, if any, are stable enough to warrant further investigation.

## 2 Materials and methods

### 2.1 Study design

We conducted an ecological country-year panel study. The unit of analysis was the country-year. The primary analytic window was 2000–2021 for trans-fat models; FAOSTAT diet-component models used 2010–2021. Reporting followed the Strengthening the Reporting of Observational Studies in Epidemiology (STROBE) statement (21) (Supplementary Figure S1).

### 2.2 Data sources

#### 2.2.1 Dietary trans-fat exposure

GBD 2021 dietary risk exposure estimates for trans-fat were used, expressed as percent energy per day (22). The GBD risk factor is defined as the mean daily intake of trans fat from all sources, mainly partially hydrogenated vegetable oils and ruminant products (23); it therefore quantifies *total* trans-fat and does not isolate the industrially produced fraction. We use the term “trans-fat exposure” throughout in this total sense, and coefficients should not be read as estimates for industrially produced TFA specifically. The source file (IHME_GBD_2021_DIET_RISK_1990_2021_TRANSFAT) contains only adult age groups; we restricted to both-sex estimates in the “% energy/day” unit and averaged across all 15 available adult age groups (25–29, 30–34, 35–39, 40–44, 45–49, 50–54, 55–59, 60–64, 65–69, 70–74, 75–79, 80–84, 85–89, 90–94, and 95 + years) for each country-year. Because this simple mean does not account for differences in age-group size, we constructed two alternatives—a population-weighted mean using World Bank age-band population counts, and an age-standardized mean using WHO World Standard Population weights (24)—and compared all three (Section 2.4; Supplementary Table S9).

#### 2.2.2 IHD and comparator outcomes

IHD and stroke outcomes were obtained from the GBD 2023 Results Tool (25, 26). The extraction settings were: measures Deaths, Incidence, and DALYs; causes ischemic heart disease, ischemic stroke, and cardiovascular diseases; metric Rate; age Age-standardized; sex Both; years 1980–2023; and all available locations (n = 204). Rates are expressed per 100,000 population. Primary outcomes were IHD incidence, IHD deaths, and IHD DALYs. Comparator outcomes were ischemic stroke incidence, deaths, and DALYs, and total CVD deaths. The corresponding settings for the trans-fat exposure extraction were: measure continuous (measure_id 19); metric rate; unit % energy/day; sex Both; the 15 adult age groups listed above; years 1990–2021. Full query settings and the country-name-to-ISO-3 mapping table are provided in the public repository (Data Availability Statement).

#### 2.2.3 Food supply proxies

Food supply data were extracted from FAOSTAT Food Balance Sheets (27). We extracted kilocalories per capita per day for major seed oils (soybean, sunflower, rapeseed/mustard, cottonseed, and maize germ oil, summed), vegetable oils, animal fats, butter/ghee, palm oil, olive oil, wheat and products, and sugar and sweeteners. FAOSTAT variables represent national food supply availability and are interpreted as dietary supply proxies.

#### 2.2.4 Policy and covariates

The WHO Global Health Observatory UHCTRANSFATS indicator provided binary trans-fat best-practice policy adoption status (28); it refers specifically to policies targeting *industrially produced* TFA and therefore does not exactly correspond to the total trans-fat exposure variable. World Bank World Development Indicators (29) provided GDP per capita (constant 2015 USD), urban population percentage, adult obesity and overweight prevalence, alcohol consumption per capita, total population (SP. POP. TOTL, used for population-weighted models), and tobacco use. The tobacco variable (SH. PRV. SMOK) is defined by its source as the prevalence of *current tobacco use* among adults aged 15 years and over—covering smoked and smokeless tobacco, age-standardized to the WHO Standard Population, both sexes combined—rather than cigarette smoking specifically; we refer to it as tobacco use throughout. Values were used exactly as published: no interpolation or carry-forward was applied to any covariate, and country-years without an observation were removed by listwise deletion within each model, which reduced the tobacco-adjusted models to 161 countries.

#### 2.2.5 Country-code harmonization

All sources were linked using ISO 3166-1 alpha-3 country codes. Country name discrepancies were resolved using a curated mapping table.

### 2.3 Exposure definitions and scaling

The primary exposure was the GBD total trans-fat estimate scaled per 0.1% point energy/day to yield interpretable coefficients. Secondary diet-component exposures were scaled per 100 kcal per capita per day: major seed oils, animal fats, butter/ghee, wheat and products, and sugar and sweeteners.

### 2.4 Statistical analysis

#### 2.4.1 Main models

Ordinary least squares (OLS) panel regression with country and year fixed effects (two-way fixed effects estimator) was used. Standard errors were clustered at the country level (30). Models were adjusted for GDP per capita (log-transformed), urbanization, alcohol consumption, and adult obesity, with all covariate values lag-matched to the exposure. The primary model specification was as follows (Equation 1) for:

$$\begin{array}{l} \mathrm{IHD}\_\text{outcome}(i,t)=\beta ₁\cdot \text{trans}\_\mathrm{fat}(i,t-\mathrm{lag})+\\ \beta ₂\cdot \log\_\mathrm{GDP}\_\mathrm{pc}(i,t-\mathrm{lag})+\\ \beta ₃\cdot \text{urbanisation}(i,t-\mathrm{lag})+\\ \beta ₄\cdot \text{alcohol}(i,t-\mathrm{lag})+\\ \beta_{5}\cdot \text{obesity}(i,t-\mathrm{lag})+\\ \text{country}\_\mathrm{FE}(i)+\text{year}\_\mathrm{FE}(t)+\varepsilon \;(i,t) \end{array}$$ (1)

Lag periods examined were 0, 3, 5, and 10 years, with 5 years pre-specified as the primary lag based on the expected time course of diet-induced atherosclerotic progression to clinically manifest IHD (6, 31). Models were estimated only when the analytic sample included at least 50 countries and 300 country-year observations after listwise deletion.

#### 2.4.2 Diet-component models

Single-component models estimated each dietary proxy separately. A core multi-component model included trans-fat, major seed oils, animal fats, wheat/products, and sugar/sweeteners simultaneously. A butter-focused model replaced animal fats with butter/ghee. To assess whether butter/ghee modified the trans-fat or policy association, interaction terms between butter/ghee and trans-fat, and between butter/ghee and WHO trans-fat policy status were estimated at 3- and 5-year lags. All diet-component models used the 5-year lag and the 2010–2021 window; the trans-fat exposure remained the GBD 2021 model-based estimate throughout. The analytic sample for diet-component models (n = 1,005; 146 countries) is smaller than the main trans-fat panel (n = 3,114; 186 countries) because complete FAOSTAT dietary data restrict the available country-years.

#### 2.4.3 Robustness: GLM estimators

To address the positive-valued, right-skewed distribution of age-standardized rates, two additional estimators were pre-specified. First, log-linear OLS used log-transformed outcomes and exponentiated coefficients, yielding approximate rate ratios (RR). Second, a Gamma GLM with a log link was estimated using the same fixed-effect design matrix (32) and clustered standard errors. Agreement across OLS, log-linear OLS, and Gamma GLM in direction and statistical significance was the pre-specified robustness criterion.

#### 2.4.4 Sensitivity analyses

Pre-specified sensitivity analyses were: (1) tobacco-use adjustment, adding the lagged national tobacco-use indicator; and (2) income stratification, estimating models separately for high-income and LMIC countries (World Bank classification). Additional comparisons used ischemic stroke and total CVD deaths as comparator outcomes.

#### 2.4.5 Robustness program

Because ecological panel estimates can depend on analytical choices that are invisible in a single headline model, the following analyses—specified before re-running the revision analyses—were added and are reported in full, regardless of direction (Supplementary Tables S9–S15).

1. *Exposure construction.* Primary models were re-estimated using the simple adult-age mean, a population-weighted mean, and a WHO-standard-population-weighted (age-standardized) mean, and the three constructions were compared directly (Supplementary Table S9).
2. *Analytical weighting.* Because unweighted models give a country-year with a small population the same influence as one with a large population, all primary models were re-estimated, weighting each observation by the outcome-year total population (Supplementary Table S10). The unweighted and weighted specifications answer different questions—the average association across countries, which are the units at which dietary policy is set, versus a population-weighted association across country-year aggregates—and both are reported (33). Because the analysis is ecological, the weighted model does not estimate an individual-level average-person effect. The precision cost of weighting was quantified using the Kish effective sample size and by computing the weighted model’s power to detect an effect of the size of the unweighted estimate.
3. *Influence.* Each primary model was reestimated 186 times, with one country excluded at a time (Supplementary Table S11).
4. *Sample composition.* Primary models were re-estimated by excluding (a) island states, defined as the 38 Small Island Developing States that are UN member states (36 of which are present in this panel), and (b) the 10 countries in the top 5% of the within-country trans-fat range (Supplementary Table S12).
5. *Effect modification.* Rather than comparing stratified subgroup estimates informally, differences by income group were tested using a fully interacted exposure × income-group model (the time-invariant income-group main effect absorbed by country fixed effects; Supplementary Table S13). Modification by country size was tested with an exposure × log-population product term. Because log-population is time-varying, its main effect is identified and was included explicitly (Supplementary Table S15).
6. *Multiplicity.* Because five dietary components were tested simultaneously in the core model, Bonferroni and Benjamini–Hochberg false discovery rate adjustments were applied to the five comparisons (Supplementary Table S13).

#### 2.4.6 Collinearity and E-values

Variance inflation factors (VIFs) were computed for the core multi-component model. E-values were computed using the VanderWeele–Ding (2017) formula (34), with point estimates converted to approximate rate ratios using the outcome mean as the reference.

All analyses used Python 3.11 (statsmodels 0.14, pandas 2.2, NumPy 1.26). All reported estimates were independently reproduced on a separate installation using the package versions specified in the public repository.

### 2.5 Ethics

This study used publicly available, aggregate country-level data without individual identifiers and did not involve individual participants. Ethical review and approval were waived by the Institutional Review Board of Seoul St. Mary’s Hospital, the Catholic University of Korea (IRB No. KC26ZISI0314).

#### 2.5.1 Generative AI statement

During the preparation of this manuscript, the author used Claude (Anthropic) and ChatGPT (OpenAI) to assist with Python code development and language editing. All statistical analyses were designed, conducted, and interpreted by the author. The author reviewed and edited all AI-assisted content and assumes full responsibility for the integrity of the research and the accuracy of the reported results.

## 3 Results

### 3.1 Data coverage

The merged panel included 8,536 country-year observations from 194 countries (1980–2023; Table 1; Supplementary Table S1). GBD trans-fat exposure was available for 6,208 country-years (194 countries, 1990–2021). GBD 2023 IHD outcomes were available for 8,536 country-years (194 countries). FAOSTAT diet variables were available for approximately 1,843 country-years (159 countries, 2010–2021). The national tobacco-use indicator was available for 164 countries in survey years. In the 5-year lag trans-fat model for IHD deaths, the analytic sample comprised 3,114 country-years from 186 countries; the analytic window begins in 2000 because alcohol consumption, a model covariate, is unavailable earlier.

**Table 1:** Characteristics of the study panel (2000–2021).

Variable	Country-years with data	Countries	Year range
GBD 2023 IHD deaths	8,536	194	1980–2023
GBD 2023 IHD incidence	6,596	194	1990–2023
GBD 2023 IHD DALYs	6,596	194	1990–2023
GBD 2021 trans-fat exposure (total)	6,208	194	1990–2021
FAOSTAT sugar/sweeteners	1,843	159	2010–2021
FAOSTAT major seed oils	1,839	159	2010–2021
FAOSTAT animal fats	1,843	159	2010–2021
FAOSTAT butter/ghee	1,843	159	2010–2021
WHO TFA policy	3,474	193	2004–2021
GDP per capita (World Bank)	6,021	191	1990–2021
Urbanization (World Bank)	6,144	192	1990–2021
Alcohol consumption (World Bank)	3,891	187	2000–2020
Adult obesity (World Bank)	6,144	192	1990–2021
Tobacco use (World Bank)	1,148	164	2000–2021

GBD, Global Burden of Disease; IHD, Ischemic heart disease; DALYs, Disability-adjusted life years; FAOSTAT, Food and Agriculture Organization Statistics; WHO, World Health Organization; TFA, Trans-fatty acids. The panel is restricted to countries with valid ISO-3166-1 alpha-3 codes (regional and global aggregates excluded). Trans-fat exposure and several World Bank covariates are available from 1990; the primary analytic sample nonetheless begins in 2000 because alcohol consumption, a model covariate, is available only from 2000 onward.

### 3.2 Trans-fat and IHD outcomes

**Figure 1:**
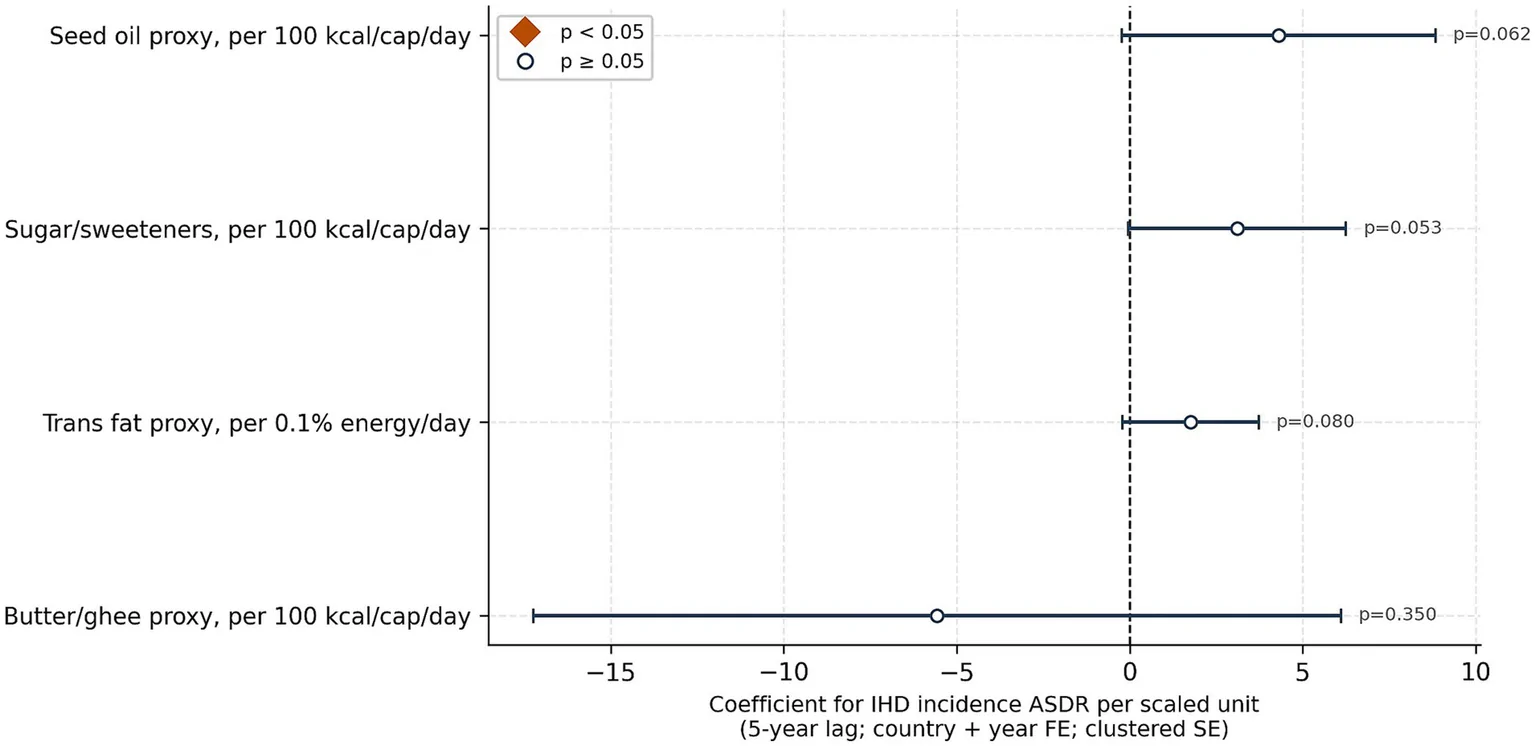
Trans-fat exposure and IHD/CVD outcomes: Country fixed-effect panel models. Associations of total trans-fat exposure with ischemic heart disease (IHD) and comparator outcomes at the 5-year lag (two-way fixed-effect OLS; *n* = 3,114 country-years; 186 countries). Coefficients represent change in age-standardized rate per 100,000 per 0.1% point of energy/day higher trans-fat exposure. Estimates for the comparator endpoints are not robust across estimators (Section 3.6) and are shown for completeness rather than as evidence of endpoint specificity. Models adjusted for four lag-matched time-varying covariates (log GDP per capita, urbanization, alcohol consumption, adult obesity); standard errors clustered at country level. DALYs: Disability-adjusted life years; CVD: Cardiovascular disease; OLS: Ordinary least squares.

Trans-fat exposure was positively associated with IHD incidence, deaths, and DALYs at 0-, 3-, and 5-year lags in country fixed-effect models (Table 2; Figure 1). The association with IHD incidence was the most consistent across the robustness program (Section 3.8) and is therefore treated as the most stable finding; the associations with deaths and DALYs are reported alongside it but proved considerably more fragile.

**Table 2:** Trans-fat exposure and cardiovascular outcomes: country fixed-effects panel models.

Outcome	Lag (years)	*β* (95% CI)a	*p*-value	*n*	Countries
IHD deaths	0	2.65 (0.57–4.73)	0.012	3,846	186
	3	3.22 (0.29–6.15)	0.031	3,480	186
	**5**	**2.74 (0.05–5.42)**	**0.046**	**3,114**	**186**
	10	1.12 (−1.17–3.42)	0.337	2,198	185
IHD incidence	0	4.24 (1.26–7.22)	0.005	3,846	186
	3	6.05 (1.31–10.79)	0.012	3,480	186
	**5**	**5.77 (1.29–10.26)**	**0.012**	**3,114**	**186**
	10	3.36 (−0.58–7.30)	0.095	2,198	185
IHD DALYs	0	50.50 (9.74–91.27)	0.015	3,846	186
	3	63.84 (9.22–118.45)	0.022	3,480	186
	**5**	**54.00 (3.90–104.10)**	**0.035**	**3,114**	**186**
	10	17.68 (−25.62–60.98)	0.424	2,198	185
Ischemic stroke deaths	5	−0.24 (−1.48–1.00)	0.704	3,114	186
Ischemic stroke incidence	5	1.02 (−0.23–2.27)	0.110	3,114	186
Ischemic stroke DALYs	5	−4.33 (−24.98–16.31)	0.681	3,114	186
CVD deaths	5	2.43 (−1.35–6.22)	0.208	3,114	186

^a^ OLS coefficient per 0.1% point energy/day higher trans-fat exposure; change in age-standardized rate per 100,000 population. All models include country fixed effects, year fixed effects, and adjustment for log GDP per capita, urbanization, alcohol consumption, and adult obesity (all lag-matched). Standard errors are clustered at the country level. Bold rows indicate the pre-specified primary lag (5 years). IHD: ischemic heart disease; DALYs: disability-adjusted life years; CVD: cardiovascular disease.

At the 5-year lag, each 0.1% point energy/day higher trans-fat exposure was associated with 5.77 higher IHD incident cases per 100,000 (95% CI: 1.29–10.26; *p* = 0.012). The corresponding estimates were 2.74 for IHD deaths (95% CI: 0.05–5.42; *p* = 0.046) and 54.00 for IHD DALYs (95% CI: 3.90–104.10; *p* = 0.035).

At the 0-year lag, trans-fat exposure was positively associated with all three IHD outcomes (incidence: *β* = 4.24, 95% CI: 1.26–7.22, *p* = 0.005; deaths: *β* = 2.65, 95% CI: 0.57–4.73, *p* = 0.012; DALYs: *β* = 50.50, 95% CI: 9.74–91.27, *p* = 0.015). At the 3-year lag, the estimates were 6.05 for incidence (95% CI: 1.31–10.79; *p* = 0.012), 3.22 for deaths (95% CI: 0.29–6.15; *p* = 0.031), and 63.84 for DALYs (95% CI: 9.22–118.45; *p* = 0.022). At the 10-year lag, associations were attenuated and non-significant for all IHD outcomes (Table 2).

### 3.3 Comparator outcomes

Trans-fat exposure was not consistently associated with ischemic stroke at the 5-year lag (deaths: *β* = −0.24, 95% CI: −1.48 to 1.00, *p* = 0.704; incidence: *β* = +1.02, 95% CI: −0.23 to 2.27, *p* = 0.110; DALYs: *β* = −4.33, 95% CI: −24.98 to 16.31, *p* = 0.681) or total CVD deaths (*β* = +2.43; 95% CI: −1.35 to 6.22; *p* = 0.208; Table 2; Figure 1). Because these comparator models were not subjected to the same robustness program and because the OLS and GLM estimators disagreed for these endpoints (Section 3.6), we do not interpret this pattern as establishing IHD-endpoint specificity.

### 3.4 Diet-component models

In single-component models for IHD incidence at 5-year lag, trans-fat was strongly positive (*β* = 7.63; 95% CI: 3.52–11.75; *p* < 0.001). Sugar/sweeteners were positive (*β* = 3.27; 95% CI: 0.21–6.33; *p* = 0.036). Major seed oils were positive but borderline (*β* = 4.01; 95% CI: −0.63 to 8.66; *p* = 0.090). Animal fats, butter/ghee, olive oil, and wheat/products were not independently positive (35–37).

**Figure 2:**
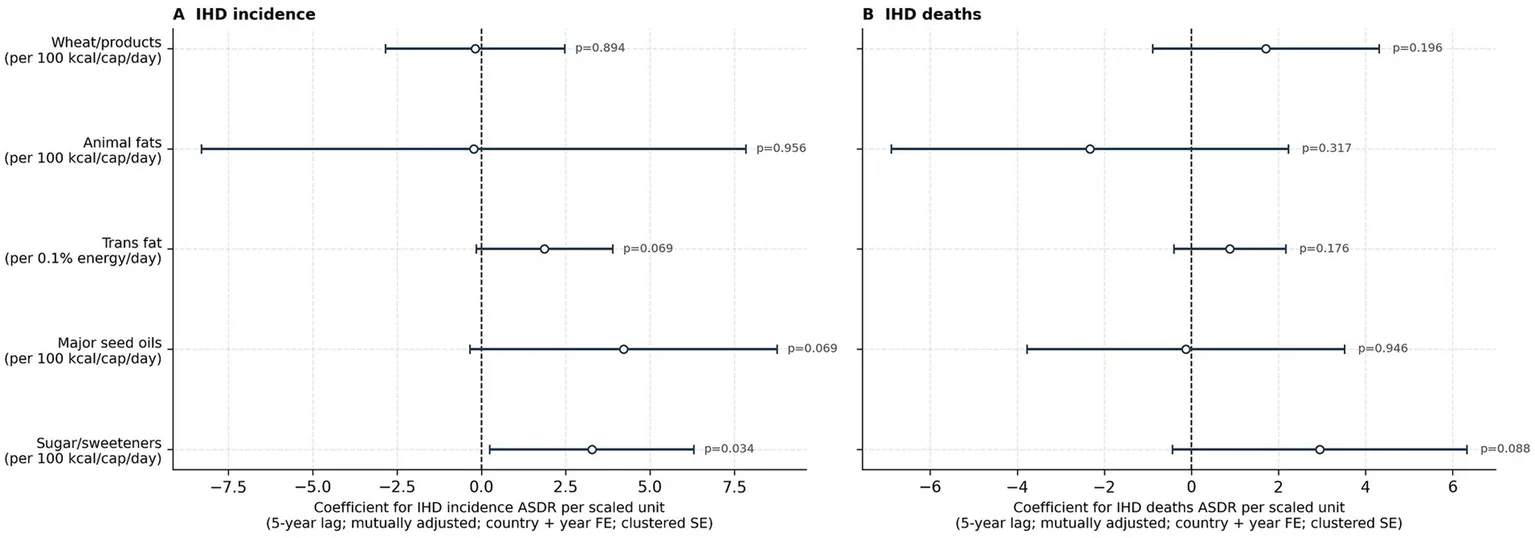
Mutually adjusted diet-component associations with IHD incidence and deaths. Diet-component associations with IHD incidence **(A)** and IHD deaths **(B)** in the core mutually adjusted model (5-year lag; two-way FE OLS; *n* = 1,005 country-years; 146 countries). Coefficients represent change in age-standardized rate per 100,000 per unit of each dietary exposure (trans-fat: Per 0.1% energy/day; all others: per 100 kcal/cap/day). All five dietary components entered simultaneously; adjusted for four lag-matched covariates; standard errors clustered at country level. *p*-values are unadjusted; no component remains significant after multiple-comparison adjustment across the five components (Supplementary Table S13). IHD: Ischemic heart disease; kcal/cap/day: Kilocalories per capita per day.

In the mutually adjusted core model (Table 3; Figure 2), the FAOSTAT sugar and sweeteners supply variable was the only component with a nominally significant association with IHD incidence (*β* = 3.27 per 100 kcal/capita/day; 95% CI: 0.25–6.29; *p* = 0.034). Trans-fat (*β* = 1.87; 95% CI: −0.15 to 3.89; *p* = 0.069) and major seed oils (*β* = 4.22; 95% CI: −0.34 to 8.77; *p* = 0.069) remained positive but borderline. Animal fats (*β* = −0.23; 95% CI: −8.29 to 7.84; *p* = 0.956) and wheat/products (*β* = −0.18; 95% CI: −2.84 to 2.47; *p* = 0.894) showed no independent positive associations.

**Table 3:** Multi-component dietary model for IHD outcomes (5-year lag, country fixed effects).

Dietary exposure (unit)	*β* (95% CI)^a^	*p*-value
Trans-fat, per 0.1% energy/day	1.87 (−0.15–3.89)	0.069
Major seed oils, per 100 kcal/cap/day	4.22 (−0.34–8.77)	0.069
Animal fats, per 100 kcal/cap/day	−0.23 (−8.29–7.84)	0.956
Wheat/products, per 100 kcal/cap/day	−0.18 (−2.84–2.47)	0.894
**Sugar/sweeteners, per 100 kcal/cap/day**	**3.27 (0.25–6.29)**	**0.034**

^a^ OLS coefficients; change in age-standardized IHD incidence rate per 100,000 population. All five diet components entered simultaneously. Model includes country fixed effects, year fixed effects, and adjustment for log GDP per capita, urbanization, alcohol consumption, and adult obesity. Standard errors clustered at country level. Bold indicates *p* < 0.05. VIF for sugar/sweeteners = 12.79; see Supplementary Table S2 for full VIF diagnostics. IHD: ischemic heart disease; VIF: variance inflation factor; kcal/cap/day: kilocalories per capita per day.

Because five components were tested simultaneously, the *p*-values were adjusted for multiplicity. After adjustment, no dietary component remained statistically significant, including sugar/sweeteners (Bonferroni *p* = 0.169; Benjamini–Hochberg FDR *p* = 0.116; Supplementary Table S13). The sugar/sweeteners estimate was also not robust to population weighting (*β* = 2.82; *p* = 0.228), although it persisted when island states were excluded (*β* = 4.42; *p* = 0.016; Supplementary Table S12). This component is therefore reported as an exploratory ecological signal rather than an established independent association. The variable measures national sugar and sweetener *supply*, which is neither added-sugar intake nor an ultra-processed food index.

### 3.5 Butter/ghee versus other dietary transition proxies

**Figure 3:**
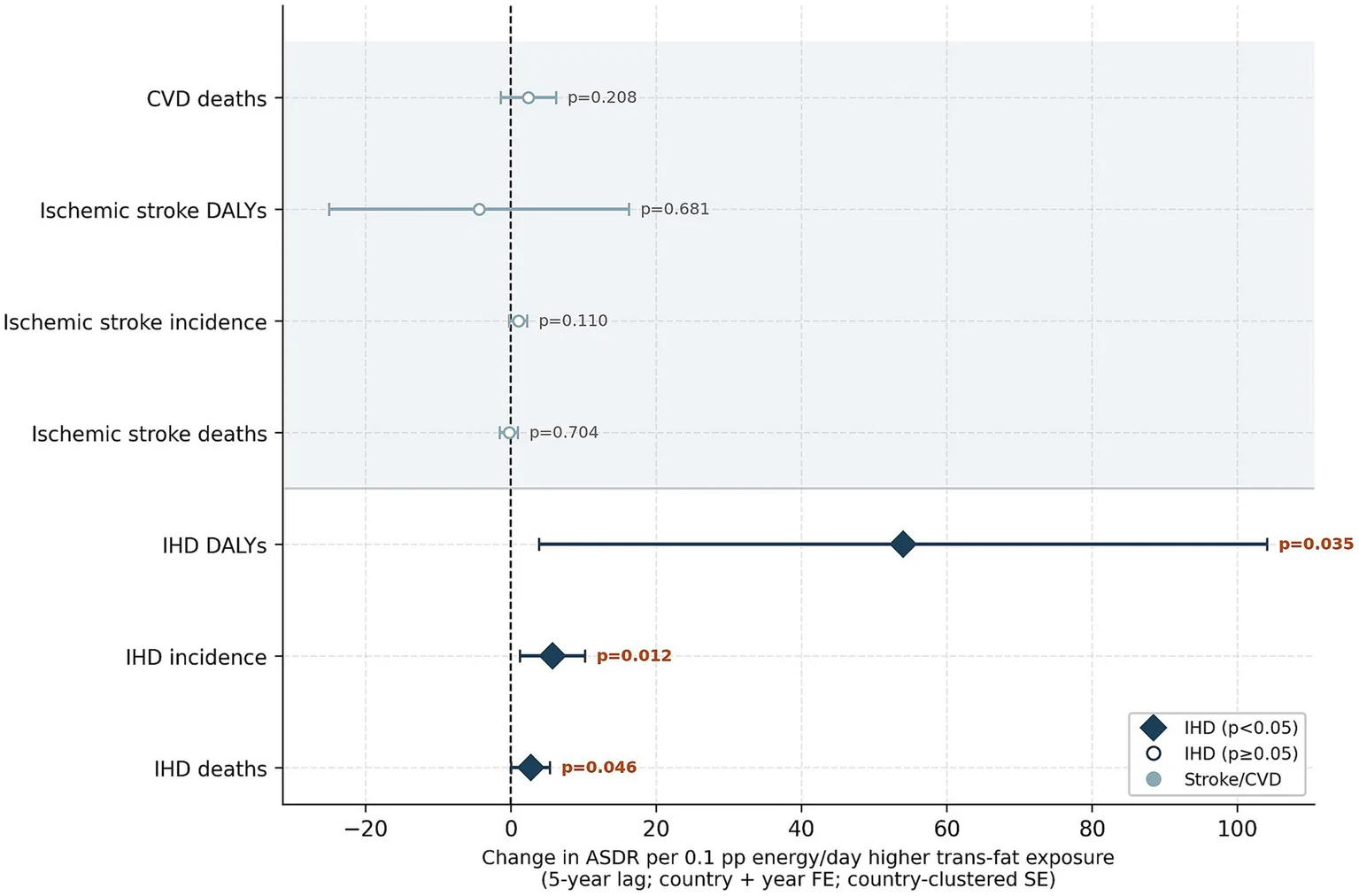
Butter/ghee versus other dietary transition proxies: Mutually adjusted model for IHD incidence. Associations of butter/ghee, trans-fat, major seed oil, and sugar/sweetener supply with IHD incidence in a mutually adjusted model (5-year lag; two-way FE OLS; *n* = 1,005 country-years; 146 countries). Coefficients represent change in age-standardized IHD incidence rate per 100,000 per unit of each exposure (trans-fat: per 0.1% point energy/day; all others: per 100 kcal/cap/day). Models include country and year fixed effects and adjustment for four lag-matched covariates (log GDP per capita, urbanization, alcohol consumption, adult obesity); standard errors clustered at the country level. Butter/ghee showed no independent harmful association. IHD: Ischemic heart disease; FE: Fixed effects; OLS: Ordinary least squares; kcal/cap/day: Kilocalories per capita per day.

In the model including butter/ghee, trans-fat, seed oils, and sugar/sweeteners for IHD incidence at 5-year lag (Table 4; Figure 3): butter/ghee showed a negative, non-significant estimate (*β* = −5.56; 95% CI: −17.23–6.11; *p* = 0.350). Trans-fat (*β* = 1.76; *p* = 0.080), seed oils (*β* = 4.31; *p* = 0.062), and sugar/sweeteners (*β* = 3.10; *p* = 0.053) all remained positive. Butter/ghee did not show a harmful interaction with trans-fat or WHO policy status.

**Table 4:** Dietary fat and sugar proxies: mutually adjusted model for IHD incidence (5-year lag).

Dietary exposure (unit)	*β* (95% CI)^a^	*p*-value
Butter/ghee, per 100 kcal/cap/day	−5.56 (−17.23–6.11)	0.350
Trans-fat, per 0.1% energy/day	1.76 (−0.21–3.73)	0.080
Major seed oils, per 100 kcal/cap/day	4.31 (−0.22–8.84)	0.062
Sugar/sweeteners, per 100 kcal/cap/day	3.10 (−0.04–6.25)	0.053

^a^ OLS coefficients; change in age-standardized IHD incidence rate per 100,000 population. Model includes country fixed effects, year fixed effects, and adjustment for log GDP per capita, urbanization, alcohol consumption, and adult obesity. Standard errors are clustered at the country level. IHD: ischemic heart disease; kcal/cap/day: kilocalories per capita per day.

### 3.6 GLM robustness check

For the association between trans-fat exposure and cardiovascular outcomes at the 5-year lag, log-linear OLS and Gamma GLM with log link produced results fully consistent with the main OLS models for all IHD outcomes (Supplementary Figure S4; Supplementary Table S7). For IHD incidence, both GLM estimators gave RR = 1.016 (95% CI: 1.004–1.028; *p* = 0.011). For IHD deaths, Gamma GLM gave RR = 1.041 (95% CI: 1.020–1.063; *p* < 0.001) and log-linear OLS gave RR = 1.042 (95% CI: 1.020–1.064; *p* < 0.001). For IHD DALYs, Gamma GLM gave RR = 1.042 (95% CI: 1.022–1.062; *p* < 0.001). All three IHD outcomes therefore met the pre-specified robustness criterion of agreement in direction and significance across all three estimators.

For the comparator endpoints, the estimators disagreed: ischemic stroke deaths (Gamma GLM: RR = 1.027; 95% CI: 1.008–1.046; *p* = 0.005) and total CVD deaths (Gamma GLM: RR = 1.027; 95% CI: 1.012–1.042; *p* < 0.001) were significant under GLM but not under OLS. By the pre-specified criterion, the stroke and total CVD associations fail the robustness check and are reported as not robust. We do not interpret the difference in rate ratios between IHD and the comparator endpoints as evidence of an IHD-dominant pattern, since doing so would require selecting the estimator that supports that reading while disregarding the criterion set out in Section 2.4. On the log scale, all five endpoints were in fact significant with rate ratios of similar magnitude (1.016–1.042), and this absence of endpoint differentiation argues against a trans-fat-specific mechanism rather than for one. It is instead consistent with the broad development-related pattern documented in Section 3.8.

### 3.7 Sensitivity analyses

#### 3.7.1 Tobacco-use adjustment

Including the lagged national tobacco-use indicator reduced the sample to 161 countries. The IHD deaths estimate remained positive and of similar magnitude (*β* = 3.60; 95% CI: 0.68–6.53; *p* = 0.016; Supplementary Figure S2; Supplementary Table S4). Estimates therefore remained broadly similar after adjustment for the available national tobacco-use indicator. Given that this indicator is available only for selected survey years, covers total tobacco use rather than cigarette smoking, and was not interpolated, this analysis cannot exclude residual confounding by smoking.

#### 3.7.2 Income group

Stratified models gave a near-null estimate in high-income countries (*n* = 60; IHD incidence: *β* = 0.32; *p* = 0.909) and a positive but non-significant estimate in LMICs (*n* = 124; *β* = 5.48; *p* = 0.133; Supplementary Figure S3; Supplementary Table S5). Because informally comparing stratified estimates cannot establish that they differ, we tested the difference formally using a fully interacted model, in which the exposure, all covariates, and the year effects are allowed to vary by income group (Supplementary Table S13). The differences were directionally consistent with the stratified pattern but none was statistically significant: IHD incidence, LMIC minus high-income = 5.15 (95% CI: −3.88 to 14.18; *p* = 0.263); IHD deaths, 0.74 (95% CI: −4.89 to 6.38; *p* = 0.796); IHD DALYs, 59.20 (95% CI: −50.40 to 168.79; *p* = 0.290). These data therefore provide no evidence of a differential association by income group, and the apparent LMIC pattern in the stratified estimates is not statistically supported.

The specification matters here. A constrained model that interacts only the exposure with income group—leaving the covariate slopes and year effects common to both groups—yields markedly different and, in our judgment, misleading difference estimates, because group differences in covariate effects are absorbed into the exposure interaction term. We therefore report the fully interacted specification, which reproduces the income-stratified estimates exactly.

### 3.8 Robustness program

#### 3.8.1 Exposure construction

The three exposure constructions were almost perfectly concordant: the simple adult-age mean correlated with the population-weighted mean at *r* = 0.9997 and with the WHO-standard-population-weighted mean at r = 0.9999 (mean absolute difference 0.025 and 0.024% points energy/day, respectively; Supplementary Table S9). Re-estimating the primary models with each construction left conclusions unchanged and slightly strengthened the estimates (IHD incidence, 5-year lag: simple mean *β* = 5.77, *p* = 0.012; population-weighted *β* = 6.32, *p* = 0.008; age-standardized *β* = 6.20, *p* = 0.009). The unweighted age aggregation therefore does not account for the observed associations.

#### 3.8.2 Analytical weighting

Weighting observations by outcome-year population produced null estimates (IHD incidence *β* = 0.12, 95% CI: −6.64 to 6.89, *p* = 0.972; IHD deaths *β* = −2.04, *p* = 0.356; IHD DALYs *β* = −33.24, *p* = 0.458; Supplementary Table S10). These estimates are underpowered and imprecise rather than evidence against the association, and we present them as such. Population weights are extremely concentrated: the five largest countries carry 47.8% of the total weight, and the Kish effective sample size—a weight-concentration diagnostic—is only 12.9 of 186 countries. The weighted analysis retains only about 39% power to detect an effect the size of the unweighted estimate (*β* = 5.77), and its confidence interval contains that estimate; it therefore neither confirms nor refutes the country-level association. Sweeping the weighting from none through log-population, square-root-population, and progressively trimmed weights to raw weights (Supplementary Table S10, Panel B) shows the estimate and its precision declining monotonically with the effective sample size rather than at any particular specification. Extending the analytic window back to 1990, the earliest year of GBD exposure data, does not materially change the effective sample, because it is limited by weight concentration rather than by the number of country-years. A formal interaction test found no evidence that the association varies with country size for IHD incidence (exposure × log-population: *p* = 0.63) or DALYs (*p* = 0.15); for IHD deaths the interaction was borderline and negative (*p* = 0.08), a non-significant hint of a weaker association in the most populous countries that is consistent with the direction of the weighting attenuation (Supplementary Table S15). The unweighted and population-weighted models answer different questions—a population-weighted association across country-year aggregates versus one giving each country equal influence—and this panel cannot distinguish them.

#### 3.8.3 Influence

In leave-one-country-out refitting, no exclusion reversed the sign of any estimate (Supplementary Table S11). The IHD incidence estimate remained statistically significant in all 186 refits (range 5.11–7.04). The IHD deaths estimate, however, fell below conventional significance in 32 of 186 refits (range 2.26–3.21), and IHD DALYs in 5 of 186. The IHD deaths association is therefore fragile to single-country exclusion, which is consistent with its borderline *p*-value (0.046) in the full sample.

#### 3.8.4 Sample composition

Excluding the 36 island states present in the panel attenuated all covariate-adjusted estimates by 39–53%, and none remained significant (IHD incidence *β* = 3.51, *p* = 0.111; deaths *β* = 1.51, *p* = 0.279; DALYs *β* = 25.28, *p* = 0.330; 150 countries; Supplementary Table S12, Panel A). Excluding the 10 countries with the largest within-country exposure swings produced a similar pattern (incidence *β* = 4.42, *p* = 0.167). Point estimates remained positive in every scenario.

Two analyses locate the source of this attenuation, and it is not the island states themselves. First, in a two-way fixed-effects model, the exposure slope is identified by within-country exposure variance, and island states supply only 1.5% of it (median within-country variance 0.035 versus 0.079 elsewhere); their exposure–outcome relationship therefore contributes little to the pooled slope, and their apparent inverse estimates in Panel A are imprecise and poorly identified. Excluding them can still move the pooled estimate by re-estimating the year effects and covariate slopes on the remaining sample, and the second analysis shows this is what happens. Adjusting covariates sequentially on the fully adjusted model’s complete-case sample (Supplementary Table S14) shows that the raw fixed-effects association is significant both in the full sample and after island exclusion (*β* = 7.35, *p* = 0.006 and *β* = 6.30, *p* = 0.020), and that the attenuation in the island-excluded sample emerges specifically when urbanization is added (p rises to 0.055) and again when obesity is added (*p* = 0.111). This is not variance inflation—once the fixed effects are applied, the covariates are not collinear (within-transformed variance inflation factors all ≤ 1.1)—but rather adjustment for development-related covariates. The reasonable reading is that the country-level trans-fat/IHD association is robust in the raw fixed-effects model but is entangled with urbanization and rising adiposity; among non-island countries, full adjustment for these development-related covariates attenuates it to non-significance. Whether this entanglement reflects confounding, mediation, or shared measurement cannot be resolved in an ecological design, but either way trans fat cannot be separated from these co-occurring features of the dietary and epidemiological transition.

### 3.9 Collinearity and E-value diagnostics

VIF analysis showed acceptable values for four of the five diet-component exposures (trans fat, major seed oils, animal fats, and wheat/products: all < 5.1); sugar/sweeteners had a higher VIF (12.79), reflecting its correlation with seed oils and wheat in the mutually adjusted model (Supplementary Table S2). The single-component coefficient was similar in magnitude (*β* = 3.27; *p* = 0.036); we do not, however, read this numerical similarity as evidence of an independent effect, because in a collinear model the estimate cannot reliably be separated from the other components of the industrial food system, and it does not survive multiple-comparison adjustment (Section 3.4; Supplementary Table S13). We therefore interpret the sugar/sweeteners variable as a marker of the broader dietary transition rather than a specific sugar effect. Socioeconomic covariates log GDP per capita (VIF = 23.0) and urbanization (VIF = 16.2) showed high VIFs in this pooled diagnostic, consistent with their strong cross-country correlation; these do not bias the diet-component estimates but preclude causal interpretation of individual covariate coefficients. We note that these pooled VIFs describe between-country correlation; within the fixed-effect (within-country) variation that actually identifies the panel coefficients, the same covariates are nearly orthogonal (within-transformed VIFs ≤ 1.1; Section 3.8), so the covariate attenuation described there reflects confounding rather than variance inflation.

E-values for 5-year lag IHD associations were: IHD deaths, E-value = 1.15 (CI lower bound 1.02); IHD incidence, 1.20 (CI lower bound 1.09); IHD DALYs, 1.16 (CI lower bound 1.04). These small values indicate that modest unmeasured confounding could explain the observed associations (Supplementary Table S3). Spearman rank correlations among diet-component exposures and covariates are reported in Supplementary Table S6.

### 3.10 Individual oil and fat types

A supplementary analysis examined all 16 individual FAOSTAT oil and fat categories as single-component exposures for IHD incidence at the 5-year lag (Supplementary Table S8; Supplementary Figures S5, S6). Soybean oil showed a significant positive association (*β* = 7.14 per 100 kcal/capita/day; 95% CI: 1.43–12.84; *p* = 0.014). Groundnut oil showed a significant inverse association (*β* = −6.97; 95% CI: −11.62 to −2.33; *p* = 0.003). Sunflower oil, rapeseed/mustard oil, palm oil, coconut oil, and total vegetable oils showed no statistically significant independent associations. Animal fats and butter/ghee remained non-significant, consistent with the main analyses.

## 4 Discussion

This country-year panel analysis found a positive association between trans-fat exposure and IHD incidence that persisted across every leave-one-country-out refit and across three ways of constructing the exposure, alongside associations for IHD deaths and DALYs that proved considerably more fragile. The lag-dependent pattern—associations at 0, 3, and 5 years with attenuation at 10 years—is compatible with the progressive accumulation of atherosclerotic disease, in which trans-fatty acids exert adverse effects on lipoprotein profiles and endothelial function that translate into clinical IHD events over years (6, 31). The direction of these estimates agrees with individual-level evidence, which is reassuring for the estimator but is not independent confirmation, since the same prior evidence motivated the analysis.

The more informative result of this study is what the robustness program revealed about where the association holds and where it breaks down. The fully adjusted estimate survives all 186 leave-one-country-out refits for IHD incidence, three exposure constructions, both OLS and Gamma GLM estimators, and lag windows from 0 to 5 years, and the raw fixed-effects estimate additionally survives exclusion of island states. What it does not survive is simultaneous full adjustment for the socioeconomic concomitants of development together with island exclusion; sequential adjustment (Section 3.8) locates this attenuation to urbanization and adiposity rather than to the island states or to collinearity. This is the substantive limit of the design: trans-fat supply, urbanization, and rising adiposity move together within countries over time, and this panel cannot attribute the IHD association to one of them rather than the bundle. We therefore present the trans-fat/IHD association as robust in its raw form and worth following up with designs that can isolate the exposure, but not as evidence that trans-fat, specifically, causes IHD at the population level.

This pattern should also be read against recent cross-sectional evidence: a GBD 2019 cross-country ecological analysis reported that higher dietary trans-fat exposure was *inversely* associated with IHD incidence, attributing this to the covariation of risk factors with socioeconomic development (38). Our within-country fixed-effects estimates carry the opposite sign—a cross-country comparison is dominated by differences in development level, whereas a within-country design removes those fixed differences but remains vulnerable to coincident development-related trends—so that the sign of the trans-fat/IHD association depends on the analytical scale, both scales remaining entangled with development. This scale-dependence is itself a demonstration of how weakly identified ecological diet–IHD associations are.

### 4.1 Interpretation of the population-weighted analysis

Population-weighted models—which, being ecological, estimate an association across country-year aggregates rather than an individual-level average-person effect—were null. It would be easy to read this as the more “representative” analysis overturning the finding, but that reading is not supported: the weighting is dominated by a handful of countries, leaving an effective sample of only 12.9 of 186 (Section 3.8; Supplementary Table S10), the weighted confidence interval comfortably contains the unweighted estimate, and the point estimate migrates toward zero as weighting intensifies, tracking the effective sample rather than any particular weighting scheme. More country-years would not resolve this, since the concentration reflects the distribution of the world’s population rather than the length of the panel. The difference between the two specifications is conventionally read as a diagnostic for effect heterogeneity rather than as evidence that either is wrong (33); a formal country-size interaction test found no significant modification for IHD incidence (Supplementary Table S15), and we did not test the equality of the two coefficients directly. The available analyses therefore neither establish a systematic size gradient nor license reading the unweighted estimate as population-averaged risk or the weighted null as refuting a country-level association.

In mutually adjusted diet-component models, the FAOSTAT sugar and sweeteners supply variable was the only nominally significant component (*p* = 0.034), but it did not survive adjustment for the five simultaneous comparisons (Bonferroni *p* = 0.169; FDR *p* = 0.116) or population weighting (*p* = 0.228). We therefore do not claim that sugar contributes independently to IHD burden. The estimate is an exploratory ecological signal that is loosely consistent with individual-level evidence linking added sugar and low carbohydrate quality to IHD risk (13, 16), and it requires confirmation with individual-level dietary measurement. It is also important not to over-read the variable itself: national sugar and sweetener supply is not added-sugar intake, refined-carbohydrate intake or an ultra-processed food index, and we avoid using those terms interchangeably. Animal fats and butter/ghee proxies did not show independent positive associations with IHD incidence after mutual adjustment.

### 4.2 Income group

Stratified estimates appeared directionally stronger in LMICs than in high-income countries, but neither group reached significance. A formal interaction test found no significant difference between the two groups for any outcome (*p* = 0.263 for IHD incidence). The differences were positive—consistent in direction with the stratified pattern—but the confidence intervals are wide and include zero comfortably. Comparing stratified estimates without testing their difference is a well-recognized error, and the correct test here supports neither a differential association nor its absence with any confidence; it simply shows the data cannot distinguish the two groups. We therefore make no claim about differential associations by income group, and these findings provide no empirical basis for prioritizing any dietary policy by country income level.

### 4.3 Seed oils as dietary transition proxies

Country-level seed oil supply is best interpreted as an industrialized food system proxy—reflecting processed food exposure, frying oil use, and dietary westernization—rather than as evidence against specific fatty acid effects (39, 40). Individual-level biomarker evidence consistently reports neutral or inverse associations between dietary linoleic acid and coronary outcomes (17, 18). Country-level seed oil supply showed a positive but borderline association with IHD incidence in single-component models; this positive ecological association is more likely to reflect ultra-processed food penetration and dietary transition rather than a direct linoleic acid effect.

### 4.4 Individual oil types

Individual FAOSTAT oil and fat categories showed heterogeneous associations with IHD incidence—soybean oil positive, groundnut oil and butter/ghee inverse for selected outcomes (Section 3.10; Supplementary Table S8; Supplementary Figures S5, S6). These divergent patterns are unlikely to reflect direct fatty acid effects—soybean and sunflower oils share similar omega-6 PUFA profiles—and do not align with clinical trial, cohort, and biomarker evidence on soybean oil, linoleic acid, and dairy fat; they more plausibly reflect food-system context (edible-oil markets, processed-food use, frying and import patterns) and residual confounding by economic development and broader dietary transition. The individual-oil results are therefore exploratory and hypothesis-generating rather than causal evidence for specific oils.

### 4.5 Butter/ghee

Butter/ghee supply did not show an independent harmful association in any model. The absence of a positive association does not establish that butter is protective: it may equally reflect limited statistical power or measurement error, since ecological country-level analyses have limited ability to disentangle the effects of individual fat sources from overall dietary patterns, particularly given the FAOSTAT butter/ghee variable’s inability to separate traditional butter from margarine or hydrogenated spreads (41, 42).

### 4.6 GLM robustness and endpoint specificity

The close agreement between OLS, log-linear OLS, and Gamma GLM for the IHD outcomes indicates that these findings are not an artifact of the OLS distributional assumption; for continuous positive rates, Gamma GLM with log link is the theoretically most appropriate estimator (32). For ischemic stroke and total CVD, however, the estimators disagreed: both were significant under GLM but not under OLS. Our pre-specified criterion required agreement across all three estimators in direction and significance, and these endpoints do not meet it. We therefore report them as not robust. In the submitted version, we interpreted the smaller GLM rate ratios for stroke and CVD (RR ≈ 1.027) relative to IHD (RR ≈ 1.041) as an IHD-dominant pattern; we no longer do so, because that reading required adopting the estimator that supported it while setting aside our robustness rule. Accordingly, this study does not establish that trans-fat associations are specific to the IHD endpoint.

### 4.7 Limitations

This is an ecological analysis; individual-level causality cannot be inferred (42). The units are countries, not people, and the analysis measures neither individual intake, nor individual outcomes, nor individual confounders—a fundamental threat to validity in ecological designs (42). FAOSTAT variables quantify national food *supply*, that is, what is available in a country rather than what is consumed, and the gap between the two is substantial; we have therefore avoided the term “intake” for these variables throughout.

Several specific limitations deserve emphasis. First, the exposure is a modeled estimate, not a measurement, and it predicts a modeled outcome. GBD dietary exposure estimates are themselves the product of statistical modeling from often sparse primary data, particularly for LMICs in earlier years, and GBD 2023 IHD outcomes are likewise modeled. Regressing one model-based quantity on another creates a risk of circularity or shared methodological bias that no sensitivity analysis in this study can rule out. Second, the exposure is total trans-fat, not industrially produced trans-fat (Section 2.2). Because ruminant TFA has not shown comparable harmful associations (6), its inclusion would be expected to bias estimates toward the null through non-differential misclassification. However, this reasoning is a conjecture about direction, not a correction, and it means our coefficients cannot be attributed to industrial TFA or read as evidence about policies targeting it. Third, E-values were small (1.15–1.20 for point estimates; CI lower bounds 1.02–1.09): an unmeasured confounder associated with both exposure and outcome by as little as 15–20% would fully explain the observed associations. In nutritional epidemiology, where diet, socioeconomic position, physical activity, healthcare quality, and tobacco use are deeply interwoven, confounding of that magnitude is not merely plausible but likely. This diagnostic alone warrants substantial caution about every association reported here. Fourth, the association cannot be separated from the broader development transition (Section 3.8): full adjustment for urbanization and adiposity, together with island exclusion, attenuates it to non-significance among non-island countries (*β* = 3.51, *p* = 0.111), and an ecological design cannot distinguish confounding from mediation or shared measurement. The IHD deaths estimate is additionally sensitive to single-country exclusion. Fifth, the population-weighted association is undetermined, since population-weighted models were null but too imprecise (about 39% power) to be decisive; the design cannot establish whether the association holds after giving greater weight to more populous countries.

Further limitations apply to specific variables. The FAOSTAT butter/ghee variable cannot separate traditional butter from margarine or hydrogenated spreads, so its coefficient is difficult to interpret. The sugar and sweeteners category aggregates heterogeneous products and does not measure added sugar. The tobacco-use indicator is available only for selected survey years, covers total tobacco use rather than cigarette smoking, and was not interpolated. The population-weighted exposure construction relies on World Bank age bands that stop at 80+, so the four oldest GBD age groups share a single weight. The panel design cannot fully account for cross-country heterogeneity in the pace of dietary transition. Finally, the multiple-comparison adjustment covered the five components of the core diet model; the wider set of exploratory analyses (multiple outcomes, lags, single-component and individual-oil models) was not jointly corrected, so those exploratory results warrant additional caution.

### 4.8 Strengths

Strengths include the use of GBD IHD-specific outcomes, integration of five global data sources, multiple dietary proxies estimated simultaneously, country and year fixed effects, clustered standard errors, and multiple lag windows. The principal strength, however, is the robustness program itself: alternative exposure constructions, population weighting, leave-one-country-out refitting, sample-composition exclusions, formal interaction testing, and multiplicity adjustment are reported in full, including where they undermine the headline estimates. All analyses reproduce exactly on an independent software environment, and the code, extraction settings, and mapping tables are publicly available. Ecological studies of this design are common in nutrition policy research, and their conclusions are rarely stress-tested this way; the fragility documented here is unlikely to be unique to this dataset.

### 4.9 Implications

These findings do not establish policy effectiveness and should not be cited as evidence for any specific intervention. The case for eliminating industrially produced trans-fat rests on individual-level trial, cohort, and biomarker evidence (6, 7), quasi-experimental evaluations of national trans-fat regulations (43, 44), and the WHO REPLACE agenda (10, 45), not on the ecological associations reported here. These associations, in any case, are based on total rather than industrial trans-fat and therefore cannot speak directly to that policy. Similarly, our sugar/sweeteners estimate does not survive multiplicity adjustment and provides no basis for sugar policy recommendations. What this analysis does offer is a prioritization signal for further research: the trans-fat/IHD incidence association was stable enough across the robustness program to warrant investigation using individual-level, biomarker-validated dietary data, standardized trans-fat measurement that separate industrial from ruminant sources, and ultra-processed food indices. It also offers a methodological caution: had we reported only the headline models, this panel would have appeared to support conclusions that its sensitivity analyses do not sustain.

In country-year fixed-effect panel models, higher trans-fat exposure was associated with higher IHD incidence, deaths, and DALYs at 3- and 5-year lags. The IHD incidence association held across leave-one-country-out refitting, three exposure constructions, and both OLS and Gamma GLM estimators. It remained significant after island-state exclusion in the raw fixed-effects specification. However, it could not be separated from the concomitants of development: among non-island countries, full adjustment for urbanization and adiposity attenuated it to non-significance (*p* = 0.111). Population-weighted models were null but too imprecise to be informative; no diet component survived multiplicity adjustment, and formal testing found no evidence for the apparent stronger association in LMICs. Given the ecological design, the modeled nature of both exposure and outcome, E-values of 1.15–1.20, and the entanglement of trans-fat supply with the broader dietary and epidemiological transition, the associations reported here are hypothesis-generating and cannot support causal or policy conclusions.

## Data Availability

All primary data sources are publicly available. GBD 2021 dietary risk exposure estimates are available at the Global Health Data Exchange (https://ghdx.healthdata.org). GBD 2023 outcomes were accessed via the GBD Results Tool (https://vizhub.healthdata.org/gbd-results). FAOSTAT Food Balance Sheets are available at https://www.fao.org/faostat. WHO trans-fat policy data are available via the WHO Global Health Observatory (https://www.who.int/data/gho). World Bank indicators are available at https://data.worldbank.org. The complete analysis code and the derived analytic datasets required to reproduce this study are openly available in a public repository (https://github.com/moonicu/transfat-ihd-country-panel), archived with a citable DOI at Zenodo (DOI: 10.5281/zenodo.21838351).
