# Supplementary material for: Dietary transition proxies and ischemic heart disease burden: a country-year panel analysis with a systematic robustness assessment
Source: Front Nutr. 2026 Aug 18;13:1883097. doi: 10.3389/fnut.2026.1883097 (PMC13529519; doi:10.3389/fnut.2026.1883097)
Supplement: Supplementary file 1 [file Data_sheet_1.DOCX]

Supplementary Material

# 1 Supplementary Tables

**Table S1. Data Coverage by Variable, Country Count, and Year Range.**

| Variable | Country-years | Countries | Year range |
| --- | --- | --- | --- |
| GBD 2023 IHD deaths | 8536 | 194 | 1980–2023 |
| GBD 2023 IHD incidence | 6596 | 194 | 1990–2023 |
| GBD 2023 IHD DALYs | 6596 | 194 | 1990–2023 |
| GBD 2021 trans-fat exposure (total) | 6208 | 194 | 1990–2021 |
| FAOSTAT sugar/sweeteners | 1843 | 159 | 2010–2021 |
| FAOSTAT major seed oils | 1839 | 159 | 2010–2021 |
| FAOSTAT animal fats | 1843 | 159 | 2010–2021 |
| FAOSTAT butter/ghee | 1843 | 159 | 2010–2021 |
| WHO TFA policy | 3474 | 193 | 2004–2021 |
| GDP per capita | 6021 | 191 | 1990–2021 |
| Urbanisation | 6144 | 192 | 1990–2021 |
| Alcohol consumption | 3891 | 187 | 2000–2020 |
| Adult obesity | 6144 | 192 | 1990–2021 |
| Tobacco use | 1148 | 164 | 2000–2021 |

*GBD: Global Burden of Disease; IHD: ischemic heart disease; DALYs: disability-adjusted life years; FAOSTAT: Food and Agriculture Organization Statistics; WHO: World Health Organization; TFA: trans-fatty acids.*

**Table S2. Variance Inflation Factors (VIF) for the Core Diet-Component Model (5-Year Lag).**

| **Variable** | **VIF** | **Interpretation** |
| --- | --- | --- |
| Trans fat (per 0.1% energy/day scaled) | 3.67 | acceptable |
| Major seed oils (per 100 kcal/cap/day scaled) | 4.71 | acceptable |
| Animal fats (per 100 kcal/cap/day scaled) | 2.89 | acceptable |
| Wheat/products (per 100 kcal/cap/day scaled) | 5.06 | VIF > 5 moderate |
| Sugar/sweeteners (per 100 kcal/cap/day scaled) | 12.79 | VIF > 10 indicates high collinearity |
| Log GDP per capita | 23.02 | VIF > 10 indicates high collinearity |
| Urbanization (%) | 16.23 | VIF > 10 indicates high collinearity |
| Alcohol (L/capita) | 4.75 | acceptable |
| Adult obesity (%) | 9.01 | VIF > 5 moderate |

*VIF computed for the mutually adjusted core model (trans-fat, major seed oils, animal fats, wheat/products, sugar/sweeteners, log GDP per capita, urbanisation, alcohol consumption, adult obesity). VIF > 10 indicates high collinearity; VIF > 5 indicates moderate collinearity.*

**Table S3. E-Values for Main IHD Trans-Fat Associations (5-Year Lag, OLS Estimates).**

| **Outcome** | **β (per 0.1% energy/day)** | **Approx. RR (point)** | **Approx. RR (CI lower)** | **E-value (point estimate)** | **E-value (CI lower bound)** |
| --- | --- | --- | --- | --- | --- |
| IHD deaths ASDR | 2.74 | 1.018 | 1.000 | 1.15 | 1.02 |
| IHD incidence ASDR | 5.77 | 1.028 | 1.006 | 1.2 | 1.09 |
| IHD DALYs ASDR | 54.00 | 1.018 | 1.001 | 1.16 | 1.04 |

*E-values computed following VanderWeele and Ding (2017). The E-value for the point estimate is the minimum strength of association (on the risk ratio scale) that an unmeasured confounder would need to have with both the exposure and outcome to fully explain away the observed association. The CI lower bound E-value applies to the lower bound of the 95% confidence interval. β: OLS coefficient (change in age-standardised rate per 100,000 per 0.1 percentage point energy/day higher trans-fat exposure). RR: approximate rate ratio derived from β and outcome mean.*

**Table S4. Sensitivity Analysis — Tobacco-Use-Adjusted Models: Trans-Fat and IHD/Comparator Outcomes (3- and 5-Year Lag).**

| **Outcome** | **Lag (years)** | **Base model β (95% CI)** | **p** | **Tobacco-use-adjusted β (95% CI)** | **p** | **N (tobacco-adj)** | **Countries** |
| --- | --- | --- | --- | --- | --- | --- | --- |
| **IHD deaths** |  |  |  |  |  |  |  |
|  | 3 | 3.22 (0.29 to 6.15) | 0.031 | 4.06 (0.99 to 7.13) | 0.009 | 799 | 161 |
|  | 5 | 2.74 (0.05 to 5.42) | 0.046 | 3.60 (0.68 to 6.53) | 0.016 | 799 | 161 |
| **IHD incidence** |  |  |  |  |  |  |  |
|  | 3 | 6.05 (1.31 to 10.79) | 0.012 | 6.54 (1.80 to 11.29) | 0.007 | 799 | 161 |
|  | 5 | 5.77 (1.29 to 10.26) | 0.012 | 6.73 (2.08 to 11.38) | 0.005 | 799 | 161 |
| **IHD DALYs** |  |  |  |  |  |  |  |
|  | 3 | 63.84 (9.22 to 118.45) | 0.022 | 79.83 (24.31 to 135.34) | 0.005 | 799 | 161 |
|  | 5 | 54.00 (3.90 to 104.10) | 0.035 | 71.65 (17.42 to 125.87) | 0.010 | 799 | 161 |
| **Ischemic stroke deaths** |  |  |  |  |  |  |  |
|  | 3 | 0.29 (-0.93 to 1.51) | 0.645 | 0.95 (-0.42 to 2.31) | 0.174 | 799 | 161 |
|  | 5 | -0.24 (-1.48 to 1.00) | 0.704 | 0.11 (-1.27 to 1.49) | 0.875 | 799 | 161 |
| **CVD deaths** |  |  |  |  |  |  |  |
|  | 3 | 3.27 (-0.70 to 7.23) | 0.106 | 4.88 (0.78 to 8.98) | 0.020 | 799 | 161 |
|  | 5 | 2.43 (-1.35 to 6.22) | 0.208 | 3.63 (-0.50 to 7.75) | 0.085 | 799 | 161 |

*β: OLS coefficient (change in age-standardised rate per 100,000 per 0.1 percentage point energy/day higher trans-fat exposure). Base model: country + year fixed effects, log GDP per capita, urbanisation, alcohol, adult obesity. Tobacco-use-adjusted model additionally includes lagged tobacco use (World Bank); sample restricted to countries with available tobacco-use data. 95% CI in parentheses; standard errors clustered at country level. IHD: ischemic heart disease; DALYs: disability-adjusted life years; CVD: cardiovascular disease.*

**Table S5. Sensitivity Analysis — Income-Stratified Models: Trans-Fat and IHD/Comparator Outcomes (3- and 5-Year Lag).**

| **Outcome** | **Lag (years)** | **All countries β (95% CI)** | **p** | **High-income β (95% CI)** | **p** | **LMIC β (95% CI)** | **p** |
| --- | --- | --- | --- | --- | --- | --- | --- |
| **IHD deaths** |  |  |  |  |  |  |  |
|  | 3 | 3.22 (0.29 to 6.15) | 0.031 | -0.27 (-3.79 to 3.24) | 0.878 | 0.48 (-4.34 to 5.30) | 0.845 |
|  | 5 | 2.74 (0.05 to 5.42) | 0.046 | -0.35 (-3.59 to 2.88) | 0.831 | 0.39 (-4.24 to 5.02) | 0.869 |
| **IHD incidence** |  |  |  |  |  |  |  |
|  | 3 | 6.05 (1.31 to 10.79) | 0.012 | 0.10 (-6.03 to 6.22) | 0.976 | 6.22 (-1.87 to 14.31) | 0.132 |
|  | 5 | 5.77 (1.29 to 10.26) | 0.012 | 0.32 (-5.24 to 5.89) | 0.909 | 5.48 (-1.67 to 12.62) | 0.133 |
| **IHD DALYs** |  |  |  |  |  |  |  |
|  | 3 | 63.84 (9.22 to 118.45) | 0.022 | -17.78 (-84.19 to 48.64) | 0.600 | 44.67 (-50.69 to 140.02) | 0.359 |
|  | 5 | 54.00 (3.90 to 104.10) | 0.035 | -20.16 (-81.30 to 40.97) | 0.518 | 39.03 (-52.22 to 130.28) | 0.402 |
| **Ischemic stroke deaths** |  |  |  |  |  |  |  |
|  | 3 | 0.29 (-0.93 to 1.51) | 0.645 | -0.76 (-2.00 to 0.49) | 0.234 | 0.06 (-2.93 to 3.04) | 0.970 |
|  | 5 | -0.24 (-1.48 to 1.00) | 0.704 | -1.19 (-2.47 to 0.09) | 0.069 | -0.06 (-3.21 to 3.09) | 0.971 |
| **CVD deaths** |  |  |  |  |  |  |  |
|  | 3 | 3.27 (-0.70 to 7.23) | 0.106 | -2.52 (-7.67 to 2.63) | 0.338 | 1.22 (-6.40 to 8.84) | 0.754 |
|  | 5 | 2.43 (-1.35 to 6.22) | 0.208 | -2.83 (-7.80 to 2.13) | 0.264 | 1.30 (-6.46 to 9.06) | 0.743 |

*β: OLS coefficient (change in age-standardised rate per 100,000 per 0.1 percentage point energy/day higher trans-fat exposure). Income classification: World Bank (2021). High-income: n = 1,140 / 60 countries (3-year lag), 1,020 / 60 (5-year lag). LMIC: n = 2,302 / 124 (3-year lag), 2,060 / 124 (5-year lag). 95% CI in parentheses; standard errors clustered at country level. IHD: ischemic heart disease; DALYs: disability-adjusted life years; CVD: cardiovascular disease; LMIC: low- and middle-income countries.*

**Table S6. Spearman Rank Correlations Among Diet-Component Exposures and Covariates (2021 Cross-Section).**

|  | **Trans fat** | **Seed oils** | **Animal fats** | **Butter/ghee** | **Sugar** | **Wheat** | **Log GDP** | **Urban %** | **Alcohol** | **Obesity %** | **Tobacco use %** |
| --- | --- | --- | --- | --- | --- | --- | --- | --- | --- | --- | --- |
| Trans fat | 1.00 | 0.16 | 0.21 | 0.19 | -0.01 | 0.16 | 0.10 | 0.27 | — | 0.14 | 0.08 |
| Seed oils | 0.16 | 1.00 | 0.59 | 0.51 | 0.44 | 0.48 | 0.58 | 0.50 | — | 0.40 | 0.18 |
| Animal fats | 0.21 | 0.59 | 1.00 | 0.87 | 0.48 | 0.51 | 0.71 | 0.46 | — | 0.44 | 0.31 |
| Butter/ghee | 0.19 | 0.51 | 0.87 | 1.00 | 0.46 | 0.54 | 0.70 | 0.44 | — | 0.39 | 0.33 |
| Sugar | -0.01 | 0.44 | 0.48 | 0.46 | 1.00 | 0.31 | 0.57 | 0.52 | — | 0.57 | 0.15 |
| Wheat | 0.16 | 0.48 | 0.51 | 0.54 | 0.31 | 1.00 | 0.45 | 0.34 | — | 0.42 | 0.39 |
| Log GDP | 0.10 | 0.58 | 0.71 | 0.70 | 0.57 | 0.45 | 1.00 | 0.68 | — | 0.51 | 0.25 |
| Urban % | 0.27 | 0.50 | 0.46 | 0.44 | 0.52 | 0.34 | 0.68 | 1.00 | — | 0.41 | 0.13 |
| Alcohol | — | — | — | — | — | — | — | — | — | — | — |
| Obesity % | 0.14 | 0.40 | 0.44 | 0.39 | 0.57 | 0.42 | 0.51 | 0.41 | — | 1.00 | 0.22 |
| Tobacco use % | 0.08 | 0.18 | 0.31 | 0.33 | 0.15 | 0.39 | 0.25 | 0.13 | — | 0.22 | 1.00 |

*Spearman rank correlations computed on the 2021 cross-section (n = 159 countries with complete FAOSTAT data). Values of 1.00 indicate perfect concordance (diagonal). — indicates data not available for that pair. All dietary variables are in kcal/capita/day except trans fat (% energy/day). GDP: gross domestic product.*

**Table S7. GLM Robustness — OLS, Log-Linear OLS, and Gamma GLM Estimates for Trans-Fat and Cardiovascular Outcomes (5-Year Lag; n = 3,114 Country-Years; 186 Countries).**

| **Outcome** | **OLS β (95% CI)** | **p** | **Log-linear OLS RR (95% CI)** | **p** | **Gamma GLM RR (95% CI)** | **p** |
| --- | --- | --- | --- | --- | --- | --- |
| **IHD deaths** | **2.74 (0.05 to 5.42)** | **0.046** | **1.042 (1.020 to 1.064)** | **<0.001** | **1.041 (1.020 to 1.063)** | **<0.001** |
| **IHD incidence** | **5.77 (1.29 to 10.26)** | **0.012** | **1.016 (1.004 to 1.028)** | **0.011** | **1.016 (1.004 to 1.028)** | **0.011** |
| **IHD DALYs** | **54.00 (3.90 to 104.10)** | **0.035** | **1.042 (1.022 to 1.063)** | **<0.001** | **1.042 (1.022 to 1.062)** | **<0.001** |
| Ischemic stroke deaths | -0.24 (-1.48 to 1.00) | 0.704 | 1.026 (1.008 to 1.045) | 0.006 | 1.027 (1.008 to 1.046) | 0.005 |
| CVD deaths | 2.43 (-1.35 to 6.22) | 0.208 | 1.027 (1.012 to 1.042) | <0.001 | 1.027 (1.012 to 1.042) | <0.001 |

*All models include country fixed effects, year fixed effects, and adjustment for log GDP per capita, urbanisation, alcohol consumption, and adult obesity (all 5-year lag-matched). Standard errors clustered at country level. OLS β: absolute rate difference per 100,000 per 0.1 percentage point energy/day higher trans-fat exposure. RR: rate ratio (exponentiated log-linear or Gamma GLM coefficient). IHD: ischemic heart disease; DALYs: disability-adjusted life years; CVD: cardiovascular disease; OLS: ordinary least squares; GLM: generalised linear model.*

**Table S8. Individual FAOSTAT Oil and Fat Types as Single-Component Exposures for IHD Outcomes (5-Year Lag; Country + Year Fixed Effects).**

| Oil/fat type | IHD incidence β (95% CI) | p | IHD deaths β (95% CI) | p | IHD DALYs β (95% CI) | p |
| --- | --- | --- | --- | --- | --- | --- |
| **Soybean oil** | **7.14 (1.43 to 12.84)** | **0.014** | **6.44 (0.75 to 12.13)** | **0.027** | **121.1 (1.5 to 240.7)** | **0.047** |
| Sunflower oil | -6.20 (-14.55 to 2.15) | 0.146 | -1.26 (-11.05 to 8.54) | 0.802 | -13.4 (-207.9 to 181.2) | 0.893 |
| Rapeseed/mustard oil | 1.74 (-6.11 to 9.59) | 0.663 | -2.40 (-10.91 to 6.10) | 0.579 | -33.4 (-173.5 to 106.8) | 0.641 |
| Palm oil | 3.29 (-2.02 to 8.59) | 0.225 | 1.00 (-4.63 to 6.62) | 0.728 | -5.0 (-130.7 to 120.6) | 0.937 |
| Palm kernel oil | 4.61 (-5.54 to 14.77) | 0.373 | 6.69 (-10.35 to 23.73) | 0.442 | 86.3 (-195.0 to 367.5) | 0.548 |
| **Groundnut oil** | **-6.97 (-11.62 to -2.33)** | **0.003** | **-5.44 (-15.17 to 4.29)** | **0.273** | **-106.3 (-294.1 to 81.4)** | **0.267** |
| Cottonseed oil | 11.55 (-25.08 to 48.18) | 0.537 | 0.43 (-52.08 to 52.94) | 0.987 | 51.3 (-1012.3 to 1114.9) | 0.925 |
| Maize germ oil | 2.56 (-15.80 to 20.92) | 0.785 | -16.56 (-34.18 to 1.05) | 0.065 | -279.6 (-636.0 to 76.7) | 0.124 |
| Olive oil | 2.59 (-11.44 to 16.62) | 0.718 | 2.91 (-8.31 to 14.13) | 0.611 | 27.4 (-215.4 to 270.1) | 0.825 |
| Sesame oil | 2.76 (-43.92 to 49.43) | 0.908 | -4.65 (-58.13 to 48.83) | 0.865 | -61.5 (-1189.1 to 1066.1) | 0.915 |
| Coconut oil | 2.97 (-4.27 to 10.22) | 0.421 | 4.64 (-2.12 to 11.40) | 0.179 | 110.9 (-32.1 to 253.8) | 0.128 |
| Other vegetable oils | -1.38 (-6.87 to 4.12) | 0.623 | -1.24 (-5.53 to 3.05) | 0.571 | -21.9 (-97.1 to 53.4) | 0.569 |
| Total vegetable oils | 1.09 (-1.87 to 4.05) | 0.471 | 0.47 (-2.01 to 2.95) | 0.711 | 9.0 (-42.6 to 60.7) | 0.732 |
| Major seed oils (sum) | 2.11 (-1.48 to 5.71) | 0.249 | 1.12 (-2.48 to 4.72) | 0.542 | 31.4 (-44.2 to 107.1) | 0.415 |
| Animal fats | 0.16 (-7.87 to 8.19) | 0.969 | -4.64 (-9.96 to 0.68) | 0.088 | -52.9 (-153.0 to 47.2) | 0.300 |
| Butter/ghee | -5.46 (-19.33 to 8.40) | 0.440 | -12.83 (-23.94 to -1.72) | 0.024 | -212.5 (-412.0 to -13.0) | 0.037 |

*β: OLS coefficient (change in age-standardised rate per 100,000 per 100 kcal/capita/day higher food supply of each oil/fat type). Each oil/fat entered as a single-component exposure (not mutually adjusted). Models include country fixed effects, year fixed effects, and adjustment for log GDP per capita, urbanisation, alcohol consumption, and adult obesity (5-year lag-matched). Standard errors clustered at country level. Bold rows indicate p < 0.05 for IHD incidence. Results are exploratory. IHD: ischemic heart disease; DALYs: disability-adjusted life years; FAOSTAT: Food and Agriculture Organization Statistics; kcal/cap/day: kilocalories per capita per day.*

**Table S9. Trans-fat exposure construction: agreement between the simple adult-age mean, a population-weighted mean, and a WHO-standard-population-weighted (age-standardised) mean, with corresponding primary models (5-year lag).**

***Panel A — agreement between constructions (n = 5,184 country-years)***

| **Comparison** | **Pearson r** | **Spearman rho** | **Mean abs. diff. (% energy/day)** | **Max abs. diff.** |
| --- | --- | --- | --- | --- |
| simple mean vs population-weighted | 0.9997 | 0.9984 | 0.025 | 0.146 |
| simple mean vs WHO-standard-weighted | 0.9999 | 0.9997 | 0.024 | 0.114 |
| population-weighted vs WHO-standard-weighted | 0.9997 | 0.9985 | 0.005 | 0.031 |

***Panel B — primary models by exposure construction (5-year lag)***

| **Exposure construction** | **Outcome** | **beta (95% CI)** | **p** | **n** | **Countries** |
| --- | --- | --- | --- | --- | --- |
| simple mean (submitted) | IHD deaths | 2.74 (0.05 to 5.42) | 0.046 | 3,114 | 186 |
| simple mean (submitted) | IHD incidence | 5.77 (1.29 to 10.26) | 0.012 | 3,114 | 186 |
| simple mean (submitted) | IHD DALYs | 54.00 (3.90 to 104.10) | 0.035 | 3,114 | 186 |
| population-weighted | IHD deaths | 3.08 (0.26 to 5.90) | 0.032 | 3,114 | 186 |
| population-weighted | IHD incidence | 6.32 (1.63 to 11.00) | 0.008 | 3,114 | 186 |
| population-weighted | IHD DALYs | 60.59 (8.19 to 112.99) | 0.023 | 3,114 | 186 |
| WHO-standard-weighted | IHD deaths | 3.03 (0.16 to 5.89) | 0.038 | 3,114 | 186 |
| WHO-standard-weighted | IHD incidence | 6.20 (1.55 to 10.86) | 0.009 | 3,114 | 186 |
| WHO-standard-weighted | IHD DALYs | 59.54 (6.53 to 112.55) | 0.028 | 3,114 | 186 |

**Table S10. Unweighted versus population-weighted models (5-year lag).**

***Panel A — primary models***

| **Model** | **Outcome** | **beta (95% CI)** | **p** | **n** | **Countries** |
| --- | --- | --- | --- | --- | --- |
| unweighted (submitted) | IHD deaths | 2.74 (0.05 to 5.42) | 0.046 | 3,114 | 186 |
| population-weighted | IHD deaths | -2.04 (-6.38 to 2.29) | 0.356 | 3,114 | 186 |
| unweighted (submitted) | IHD incidence | 5.77 (1.29 to 10.26) | 0.012 | 3,114 | 186 |
| population-weighted | IHD incidence | 0.12 (-6.64 to 6.89) | 0.972 | 3,114 | 186 |
| unweighted (submitted) | IHD DALYs | 54.00 (3.90 to 104.10) | 0.035 | 3,114 | 186 |
| population-weighted | IHD DALYs | -33.24 (-120.95 to 54.47) | 0.458 | 3,114 | 186 |
| unweighted (submitted) | Ischemic stroke deaths | -0.24 (-1.48 to 1.00) | 0.704 | 3,114 | 186 |
| population-weighted | Ischemic stroke deaths | -2.32 (-5.87 to 1.23) | 0.201 | 3,114 | 186 |
| unweighted (submitted) | CVD deaths | 2.43 (-1.35 to 6.22) | 0.208 | 3,114 | 186 |
| population-weighted | CVD deaths | -7.44 (-18.72 to 3.84) | 0.196 | 3,114 | 186 |

***Panel B — weighting intensity, information loss, and power (IHD incidence, 5-year lag)***

| **Weighting** | **beta (95% CI)** | **p** | **Effective countries** | **Power to detect beta = 5.77** |
| --- | --- | --- | --- | --- |
| unweighted | 5.77 (1.29 to 10.26) | 0.012 | 186 | 71% |
| log(population) | 5.39 (0.93 to 9.84) | 0.018 | 183 | 72% |
| sqrt(population) | 2.81 (-2.29 to 7.91) | 0.280 | 80 | 60% |
| population, trimmed at 75th percentile | 2.83 (-2.43 to 8.10) | 0.291 | 103 | 58% |
| population, trimmed at 90th percentile | 3.09 (-2.87 to 9.05) | 0.310 | 74 | 48% |
| population, trimmed at 95th percentile | 2.75 (-3.79 to 9.29) | 0.410 | 58 | 41% |
| population, trimmed at 99th percentile | 0.19 (-6.59 to 6.97) | 0.956 | 14 | 39% |
| population (untrimmed) | 0.12 (-6.64 to 6.89) | 0.972 | 13 | 39% |

*Population weights are outcome-year total population (World Bank SP.POP.TOTL); Panels A and B use the same weighting time. As weighting concentrates influence in a few large countries, the effective sample (Kish weight-concentration diagnostic) falls from 186 to 12.9 and power to detect the unweighted effect is about 39%. The weighted analysis is therefore underpowered and its confidence interval contains the unweighted estimate, so it neither confirms nor refutes the country-level association. Extending the window to 1990 does not materially change the effective sample, as it is limited by weight concentration (five countries hold 47.8% of the weight), not the number of country-years. Whether the slope varies with country size is tested in Table S15.*

**Table S11. Leave-one-country-out analysis for the primary models (5-year lag).**

| **Outcome** | **Full-sample beta** | **beta range across refits** | **Refits** | **Refits losing significance** | **Sign reversals** |
| --- | --- | --- | --- | --- | --- |
| IHD DALYs | 54.00 | 45.20 to 61.51 | 186 | 5 | 0 |
| IHD deaths | 2.74 | 2.26 to 3.21 | 186 | 32 | 0 |
| IHD incidence | 5.77 | 5.11 to 7.04 | 186 | 0 | 0 |

*Each model was re-estimated 186 times, excluding one country at a time. No refit reversed the sign. IHD incidence retained significance in every refit; IHD deaths did not in 32 of 186, indicating sensitivity to single-country exclusion.*

**Table S12. Sensitivity of the primary models to sample composition (5-year lag).**

***Panel A — covariate-adjusted models***

| **Sample** | **Outcome** | **beta (95% CI)** | **p** | **n** | **Countries** |
| --- | --- | --- | --- | --- | --- |
| Full sample | IHD deaths | 2.74 (0.05 to 5.42) | 0.046 | 3,114 | 186 |
| Full sample | IHD incidence | 5.77 (1.29 to 10.26) | 0.012 | 3,114 | 186 |
| Full sample | IHD DALYs | 54.00 (3.90 to 104.10) | 0.035 | 3,114 | 186 |
| Excluding UN SIDS island states | IHD deaths | 1.51 (-1.22 to 4.25) | 0.279 | 2,502 | 150 |
| Excluding UN SIDS island states | IHD incidence | 3.51 (-0.80 to 7.83) | 0.111 | 2,502 | 150 |
| Excluding UN SIDS island states | IHD DALYs | 25.28 (-25.55 to 76.11) | 0.330 | 2,502 | 150 |
| UN SIDS island states only | IHD deaths | -49.90 (-132.88 to 33.08) | 0.239 | 612 | 36 |
| UN SIDS island states only | IHD incidence | -65.92 (-119.77 to -12.08) | 0.016 | 612 | 36 |
| UN SIDS island states only | IHD DALYs | -883.25 (-2575.32 to 808.83) | 0.306 | 612 | 36 |
| Excluding largest exposure swings (top 5%) | IHD deaths | 2.44 (-1.98 to 6.87) | 0.280 | 2,944 | 176 |
| Excluding largest exposure swings (top 5%) | IHD incidence | 4.42 (-1.86 to 10.70) | 0.167 | 2,944 | 176 |
| Excluding largest exposure swings (top 5%) | IHD DALYs | 40.10 (-45.19 to 125.39) | 0.357 | 2,944 | 176 |

***Panel B — same exclusion without covariate adjustment (fixed effects only)***

| **Sample** | **Outcome** | **beta (95% CI)** | **p** | **n** | **Countries** |
| --- | --- | --- | --- | --- | --- |
| Full sample | IHD deaths | 4.61 (1.18 to 8.04) | 0.008 | 4,268 | 194 |
| Full sample | IHD incidence | 7.31 (0.98 to 13.65) | 0.024 | 4,268 | 194 |
| Full sample | IHD DALYs | 89.24 (20.11 to 158.37) | 0.011 | 4,268 | 194 |
| Excluding UN SIDS island states | IHD deaths | 4.14 (0.80 to 7.49) | 0.015 | 3,432 | 156 |
| Excluding UN SIDS island states | IHD incidence | 6.57 (0.21 to 12.93) | 0.043 | 3,432 | 156 |
| Excluding UN SIDS island states | IHD DALYs | 75.90 (8.64 to 143.15) | 0.027 | 3,432 | 156 |

*Island states are the 36 Small Island Developing States in the panel. In the adjusted models (Panel A) their exclusion attenuates estimates by 39-53% and none stays significant, but they supply only 1.5% of the identifying within-country exposure variance, and without covariate adjustment (Panel B) all three outcomes remain significant. The attenuation reflects covariate adjustment (Table S14), not island states driving the association; their Panel A estimates are correspondingly imprecise and poorly identified.*

**Table S13. Formal income-group interaction test and multiple-comparison adjustment.**

***Panel A — trans-fat x income-group interaction (fully interacted model, 5-year lag)***

| **Outcome** | **High-income slope** | **LMIC slope** | **LMIC - HIC difference (95% CI)** | **Interaction p** |
| --- | --- | --- | --- | --- |
| IHD deaths | -0.35 | 0.39 | 0.74 (-4.89 to 6.38) | 0.796 |
| IHD incidence | 0.32 | 5.48 | 5.15 (-3.88 to 14.18) | 0.263 |
| IHD DALYs | -20.16 | 39.03 | 59.20 (-50.40 to 168.79) | 0.290 |

*The exposure, all covariates, and year effects vary by income group; country fixed effects absorb the income-group main effect. This reproduces the stratified estimates exactly. No difference is significant, so there is no evidence of a differential association by income group.*

***Panel B — multiple-comparison adjustment, five core diet components (IHD incidence, 5-year lag)***

| **Dietary exposure** | **beta** | **Unadjusted p** | **Bonferroni p** | **Benjamini-Hochberg FDR p** |
| --- | --- | --- | --- | --- |
| Trans-fat (per 0.1% energy/day) | 1.87 | 0.069 | 0.347 | 0.116 |
| Major seed oils (per 100 kcal/cap/day) | 4.22 | 0.069 | 0.347 | 0.116 |
| Animal fats (per 100 kcal/cap/day) | -0.23 | 0.956 | 1.000 | 0.956 |
| Wheat/products (per 100 kcal/cap/day) | -0.18 | 0.894 | 1.000 | 0.956 |
| Sugar/sweeteners (per 100 kcal/cap/day) | 3.27 | 0.034 | 0.169 | 0.116 |

*No dietary component remains significant after adjustment for multiple comparisons.*

**Table S14. Sequential covariate adjustment for IHD incidence (5-year lag), full sample versus island-excluded, on an identical complete-case sample.**

| **Adjustment** | **Full sample beta** | **Full p** | **Island-excluded beta** | **Island-excluded p** |
| --- | --- | --- | --- | --- |
| fixed effects only | 7.35 | 0.006 | 6.30 | 0.020 |
| + log GDP per capita | 6.96 | 0.005 | 6.11 | 0.015 |
| + urbanisation | 6.25 | 0.010 | 4.72 | 0.055 |
| + alcohol | 6.16 | 0.011 | 4.65 | 0.059 |
| + obesity (full model) | 5.77 | 0.012 | 3.51 | 0.111 |

*Both columns use the complete-case sample of the fully adjusted model, so steps differ only in adjustment. The raw fixed-effects association is significant in both samples; the island-excluded attenuation appears after adjustment for urbanisation and then obesity, and is not accompanied by high within-model collinearity (within-transformed VIFs trans-fat = 1.02, log_gdp_pc = 1.10, urban_pop_pct = 1.04, alcohol_liters_pc = 1.09, obesity_adults_pct = 1.04). This pattern is consistent with the association being entangled with development-related covariates rather than with variance inflation, though an ecological design cannot distinguish confounding from mediation or shared measurement. Either way, trans fat cannot be separated from co-occurring features of the dietary and epidemiological transition.*

**Table S15. Country-size effect modification: exposure x log-population interaction (5-year lag).**

| **Outcome** | **Slope at mean size** | **p** | **Interaction / unit log-pop (95% CI)** | **Interaction p** |
| --- | --- | --- | --- | --- |
| IHD deaths | 2.62 | 0.061 | -1.37 (-2.87 to 0.14) | 0.075 |
| IHD incidence | 5.26 | 0.024 | -0.58 (-2.92 to 1.76) | 0.625 |
| IHD DALYs | 52.02 | 0.049 | -21.84 (-51.27 to 7.59) | 0.146 |

*log(population) is time-varying within countries, so its main effect is included alongside the interaction; the exposure coefficient is the slope at mean (centred) log-population. There is no significant modification by country size for IHD incidence (p = 0.63) or DALYs (p = 0.15); for IHD deaths the interaction is borderline and negative (p = 0.08), a non-significant hint of a weaker association in the most populous countries, consistent with the attenuation under population weighting (Table S10). At mean country size the slope remains positive for all three outcomes.*

# 2 Supplementary Figures

**Figure S1.** Study data flow and analytic design (STROBE diagram). Data flow and analytic design diagram (STROBE). Numbers indicate country-years available at each stage of data linkage and analytic restriction.

**Figure S2.** Tobacco-use-adjusted sensitivity analysis: trans-fat/IHD associations. Sensitivity analysis: tobacco-use-adjusted versus base models for trans-fat/IHD associations (5-year lag; two-way FE OLS; n = 161 countries with available tobacco-use data). Coefficients represent change in age-standardised rate per 100,000 per 0.1 percentage point energy/day higher trans-fat exposure. IHD: ischemic heart disease; DALYs: disability-adjusted life years.

**Figure S3.** Income-stratified models: trans-fat/IHD incidence by high-income vs. LMIC. Sensitivity analysis: income-stratified models for trans-fat/IHD incidence associations (5-year lag; two-way FE OLS; high-income: n = 60 countries; LMIC: n = 124 countries). Coefficients represent change in age-standardised IHD incidence rate per 100,000 per 0.1 percentage point energy/day higher trans-fat exposure. LMIC: low- and middle-income countries; IHD: ischemic heart disease.

**Figure S4.** OLS versus Gamma GLM robustness comparison for trans-fat/CVD associations. GLM robustness comparison for trans-fat/cardiovascular outcome associations (5-year lag; n = 3,114 country-years; 186 countries). Left panel: OLS coefficients (absolute rate differences per 100,000); right panel: rate ratios from log-linear OLS and Gamma GLM with log link. IHD: ischemic heart disease; DALYs: disability-adjusted life years; CVD: cardiovascular disease; OLS: ordinary least squares; GLM: generalised linear model.

**Figure S5.** Individual FAOSTAT fat and oil types as single-component exposures for IHD incidence. Individual FAOSTAT oil and fat types as single-component exposures for IHD incidence (5-year lag; country + year fixed effects). Coefficients represent change in age-standardised IHD incidence rate per 100,000 per 100 kcal/cap/day higher dietary supply. Horizontal bars represent 95% confidence intervals. Results are exploratory and hypothesis-generating. IHD: ischemic heart disease; FAOSTAT: Food and Agriculture Organization Statistics; kcal/cap/day: kilocalories per capita per day.

**Figure S6.** P-value heatmap: FAOSTAT fat/oil exposures across IHD outcomes. P-value heatmap for individual FAOSTAT oil and fat exposures across IHD outcomes (IHD deaths, incidence, DALYs) at the 5-year lag (country + year fixed effects). Colour intensity reflects −log10(p-value); cells are labelled with the direction of the coefficient (+ or −). Results should be interpreted as exploratory. IHD: ischemic heart disease; DALYs: disability-adjusted life years.
